# BIOLOGICAL AGE AND COGNITIVE PERFORMANCE AMONG LATE MID-LIFE WOMEN

**DOI:** 10.1093/geroni/igad104.2096

**Published:** 2023-12-21

**Authors:** Rebecca Thurston, Cynthia Kusters, Yuefang Chang, Pauline Maki, Judith Carroll

**Affiliations:** University of Pittsburgh, Pittsburgh, Pennsylvania, United States; University of California, Los Angeles, Los Angeles, California, United States; University of Pittsburgh, Pittsburgh, Pennsylvania, United States; University of Illinois at Chicago, Chicago, Illinois, United States; University of California, Los Angeles, Los Angeles, California, United States

## Abstract

Older biologic age, indicated by accelerated epigenetic aging, may place women at risk for poorer cognitive performance as they age. However, findings are inconsistent and based upon limited neuropsychological batteries. Studies of midlife women are few, yet women comprise two thirds of those with Alzheimer’s disease, and midlife is a critical transition and potential window for early intervention. We tested whether greater epigenetic age would be associated with poorer cognitive performance among late midlife women. 224 MsBrain study participants (average age=59 years, 82% White, 14% Black, 4% other ethnicities) underwent phlebotomy, including epigenetic aging clock estimates (GrimAge, DunedinPACE, PCPhenoAGE), and a neuropsychological battery assessing global cognitive function (Montreal Cognitive Assessment), verbal learning and memory (California Verbal Learning Test), working memory and attention (Letter Number Sequencing), letter fluency (P, R, W); semantic fluency (animal task), spatial ability (Card Rotations), processing speed (Symbol Digit Modalities Test), and perceptual speed (Finding A’s). In linear regression models controlling for age, race, and education, older PCPhenoAge was associated with poorer global cognitive performance [B(SE)=-.07(.03), p=.01], verbal memory [B(SE)=-.09(.03), p=.006], working memory [B(SE)=-.12(.03), p=.0001], attention [B(SE)=-.10(.04), p=.02], spatial ability [B(SE)=-.77(.34), p=.02], processing speed [B(SE)=-.24(.09), p=.007], and somewhat lower letter fluency [B(SE)=-.23(.13), p=.07]. GrimAge and DunedinPACE showed similar yet less consistent patterns. Interactions by race (p’s<.05) indicate that associations between older biologic age and poorer cognitive performance (verbal learning, verbal memory, processing speed) was particularly pronounced among Black women in the sample. Older biologic age is associated with poorer cognitive performance across multiple domains among late midlife women, particularly for Black women. Biologic age may serve as an early indicator of risk that could help identify women warranting early intervention to help maintain cognitive function. Supported by NIH RF1AG053504, R01AG053504, K24HL123565

